# Durability and expansion of neutralizing antibody breadth following Ad26.COV2.S vaccination of mice

**DOI:** 10.1038/s41541-022-00454-4

**Published:** 2022-02-23

**Authors:** Shant H. Mahrokhian, Lisa H. Tostanoski, Catherine Jacob-Dolan, Roland C. Zahn, Frank Wegmann, Katherine McMahan, Jingyou Yu, Makda S. Gebre, Esther A. Bondzie, Huahua Wan, Olivia Powers, Tianyi Ye, Julia Barrett, Hanneke Schuitemaker, Dan H. Barouch

**Affiliations:** 1 Center for Virology and Vaccine Research, Beth Israel Deaconess Medical Center, Harvard Medical School, Boston, MA 02215 USA; 2 Tufts University School of Medicine, Boston, MA 02111 USA; 3 Harvard Medical School, Boston, MA 02115 USA; 4 Ragon Institute of MGH, MIT, and Harvard, Cambridge, MA 02139 USA; 5 Janssen Vaccines & Prevention BV, Leiden, Netherlands; 6 Massachusetts Consortium on Pathogen Readiness, Boston, MA 02115 USA

**Keywords:** Viral infection, Vaccines

## Abstract

Emerging SARS-CoV-2 variants with the potential to escape binding and neutralizing antibody responses pose a threat to vaccine efficacy. We recently reported expansion of broadly neutralizing activity of vaccine-elicited antibodies in humans 8 months following a single immunization with Ad26.COV2.S. Here, we assessed the 15-month durability of antibody responses and their neutralizing capacity to B.1.617.2 (delta) and B.1.351 (beta) variants following a single immunization of Ad26.COV2.S in mice. We report the persistence of binding and neutralizing antibody titers following immunization with a concomitant increase in neutralizing antibody breadth to delta and beta variants over time. Evaluation of bone marrow and spleen at 15 months postimmunization revealed that Ad26.COV2.S-immunized mice tissues contained spike-specific antibody-secreting cells. We conclude that immunization with Ad26.COV2.S elicits a robust immune response against SARS-CoV-2 spike, which expands over time to neutralize delta and beta variants more robustly, and seeds bone marrow and spleen with long-lived spike-specific antibody-secreting cells. These data extend previous findings in humans and support the use of a mouse model as a potential tool to further explore the dynamics of the humoral immune response following vaccination with Ad26.COV2.S.

## Introduction

Several vaccines have been developed against severe acute respiratory syndrome coronavirus 2 (SARS-CoV-2)^1–4^, the virus responsible for the coronavirus disease 2019 (COVID-19) pandemic^5,6^. Induction of neutralizing antibodies by COVID-19 vaccines has proven an important correlate of protection from SARS-CoV-2 infection in several animal models^7–14^. However, numerous variants of concern have emerged with the potential to evade vaccine-induced polyclonal neutralizing antibody responses or those raised by natural infection^15–18^. Although vaccines remain robustly protective against severe disease, hospitalization, and death, emerging variants may diminish vaccine efficacy against infection or mild disease^19–22^. This has prompted evaluation of booster immunizations^23,24^, especially given that vaccine-elicited antibodies wane over time^25,26^. Of note, the B.1.351 (beta) variant first identified in South Africa in December 2020^27^, and the B.1.617.2 (delta) variant first identified in India in April 2021^28^, have caused substantial morbidity and mortality worldwide, the latter establishing itself as the dominant source of COVID-19 disease in the United States in July 2021.

The immunogen encoding the full-length spike protein and a di-proline mutation stabilizing prefusion conformation (S.PP), has previously demonstrated robust induction of protective binding and neutralizing antibody responses in both animals^9–11,29^ and humans^30–32^. The replication-incompetent adenovirus serotype 26 (Ad26)^33–35^ expressing the S.PP immunogen, termed Ad26.S.PP or Ad26.COV2.S, was authorized for emergency use in the United States after demonstrating protective efficacy in a global phase 3 clinical trial in early 2021^4^. More recently, it was shown in humans that Ad26.COV2.S elicits expansion of neutralization breadth by 8 months following immunization^36^.

In this study, we assessed the immunogenicity and 15-month durability of vaccine-elicited humoral immune responses in mice immunized with Ad26.COV2.S. We also compared the immunogenicity of Ad26.COV2.S to an Ad26 vector expressing a soluble version of the S.PP immunogen via deletion of the transmembrane domain and cytoplasmic tail (Ad26.S.dTM.PP). Vaccine doses were titrated to evaluate differences in immunogenicity between candidates at suboptimal doses. Furthermore, the long-term kinetics of antibody binding and neutralizing capacity to SARS-CoV-2 variants of concern were assessed in Ad26.COV2.S-immunized mice. We demonstrate the significant expansion of antibody breadth and durability of humoral responses over time following a single immunization of Ad26.COV2.S.

## Results

### Immunogenicity of Ad26.COV2.S and Ad26.S.dTM.PP

**Fig. 1:**
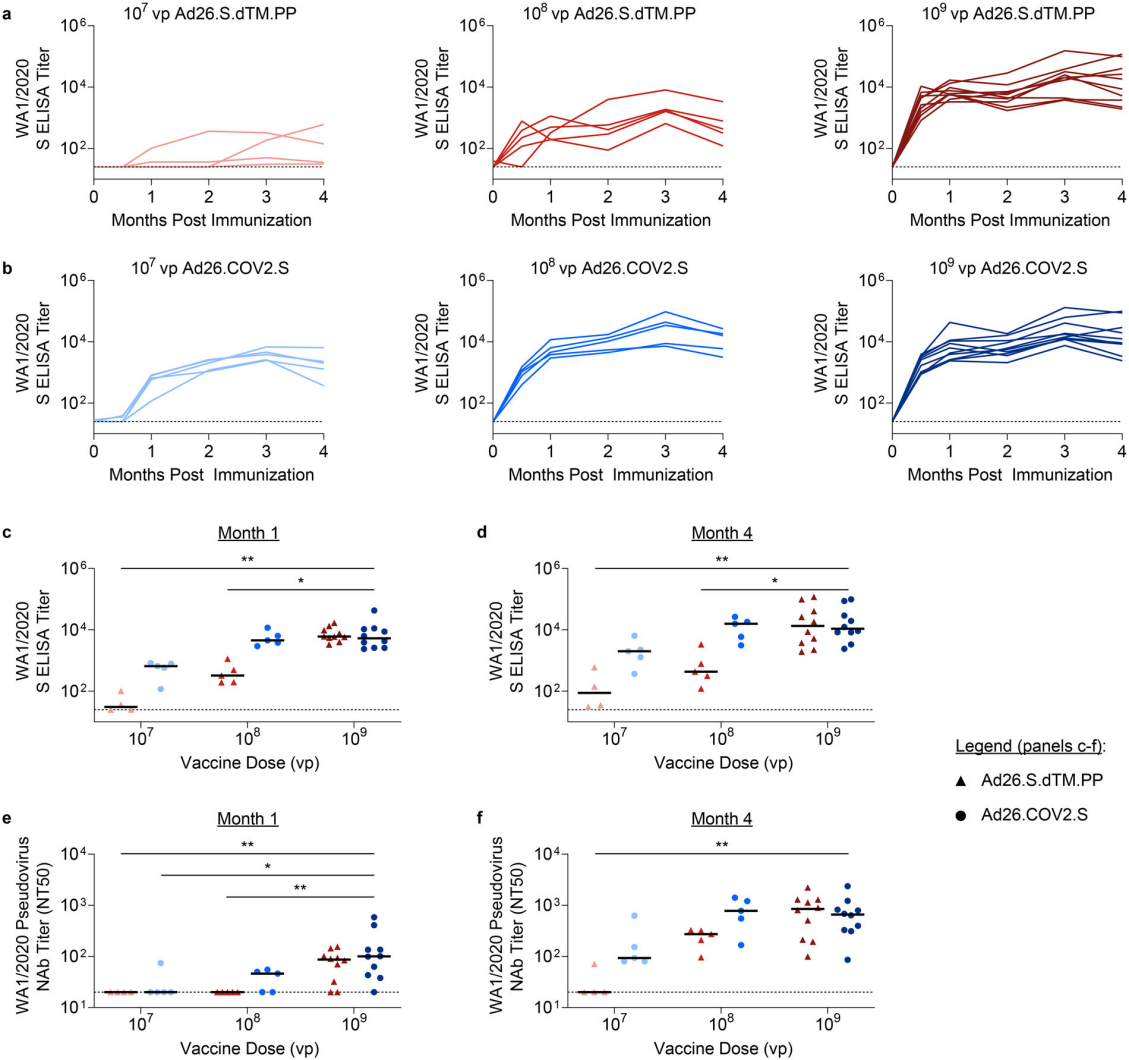
Comparison of humoral responses elicited by two candidate Ad26 vaccines. Binding antibody responses were assessed by WA1/2020 S-specific ELISA at weeks 0, 2, 4, 8, 12, and 16 in mice immunized with 1 × 10^7^ (*N* = 5), 1 × 10^8^ (*N* = 5), or 1 × 10^9^ (*N* = 10) viral particles (vp) of (**a**) Ad26.S.dTM.PP or (**b**) Ad26.COV2.S. Binding antibody responses of Ad26.S.dTM.PP (triangles) and Ad26.COV2.S (circles) immunized mice are displayed at (**c**) 1 month and (**d**) 4 months postimmunization. Similarly, neutralizing activity of vaccine-elicited antibody responses are displayed as the 50% neutralization titer (NT50) at (**e**) 1 month and (**f**) 4 months postimmunization. Horizontal bars reflect median titers. Dotted lines reflect assay limit of quantification. P values reflect Kruskal Wallis tests with Dunn’s multiple comparisons (*P < 0.05; **P < 0.01; ***P < 0.001).

To compare the immunogenicity of two candidate Ad26 vaccines expressing modified versions of the SARS-CoV-2 full-length spike (S) protein, groups of female wild-type BALB/c mice were immunized with a single dose of 1 × 10^7^ (low dose) (*N* = 5), 1 × 10^8^ (middle dose) (*N* = 5), or 1 × 10^9^ (high dose) (*N* = 10) viral particles (vp) Ad26.COV2.S or Ad26.S.dTM.PP via the intramuscular (IM) route. Peripheral blood was collected at indicated timepoints to quantify serum antibody responses over time. Evaluation of binding antibody titers by enzyme-linked immunosorbent assay (ELISA) showed induction of WA1/2020 S-specific titers in mice immunized with Ad26.S.dTM.PP, though responses appeared markedly diminished with the low dose (Fig. 1a). Immunization with Ad26.COV2.S yielded robust WA1/2020 S-specific binding antibody titers at all tested doses, although responses were delayed with the low dose (Fig. 1b).

At 1 month and 4 months postimmunization, WA1/2020 S-specific binding titers and neutralizing antibody (NAb) responses induced by each vaccine regimen were compared to those induced by the high dose Ad26.COV2.S. Binding titers elicited by Ad26.COV2.S were similar across all three doses, though titers from the low dose appeared to trend lower (Fig. 1c, d). In contrast, binding titers elicited by Ad26.S.dTM.PP were significantly reduced in the low dose (*P* = 0.002) and middle dose (*P* = 0.028) at 1 month (Fig. 1c) and 4 months postimmunization (*P* = 0.002 and *P* = 0.012, respectively) (Fig. 1d). NAb responses were assessed by a lentiviral pseudovirus neutralization assay as described previously^37^. At 1 month postimmunization, low dose vaccination with Ad26.COV2.S elicited significantly reduced NAb titers compared to high dose vaccination (*P* = 0.045) (Fig. 1e). By month 4, however, all mice immunized with the low dose Ad26.COV2.S displayed detectable and statistically similar NAb titers to the high dose (Fig. 1f). Conversely, 1 month postimmunization NAb titers were significantly reduced and undetectable in all mice immunized with Ad26.S.dTM.PP at either a middle (*P* = 0.007) or low dose (*P* = 0.015) (Fig. 1e), with low dose NAb titers still being undetectable in 3 mice at 4 months postimmunization (*P* = 0.005) (Fig. 1f). Taken together, these data suggest that deletion of the transmembrane domain from the S.PP immunogen impairs vaccine immunogenicity in mice.

### Antibody binding responses to SARS-CoV-2 variants

**Fig. 2:**
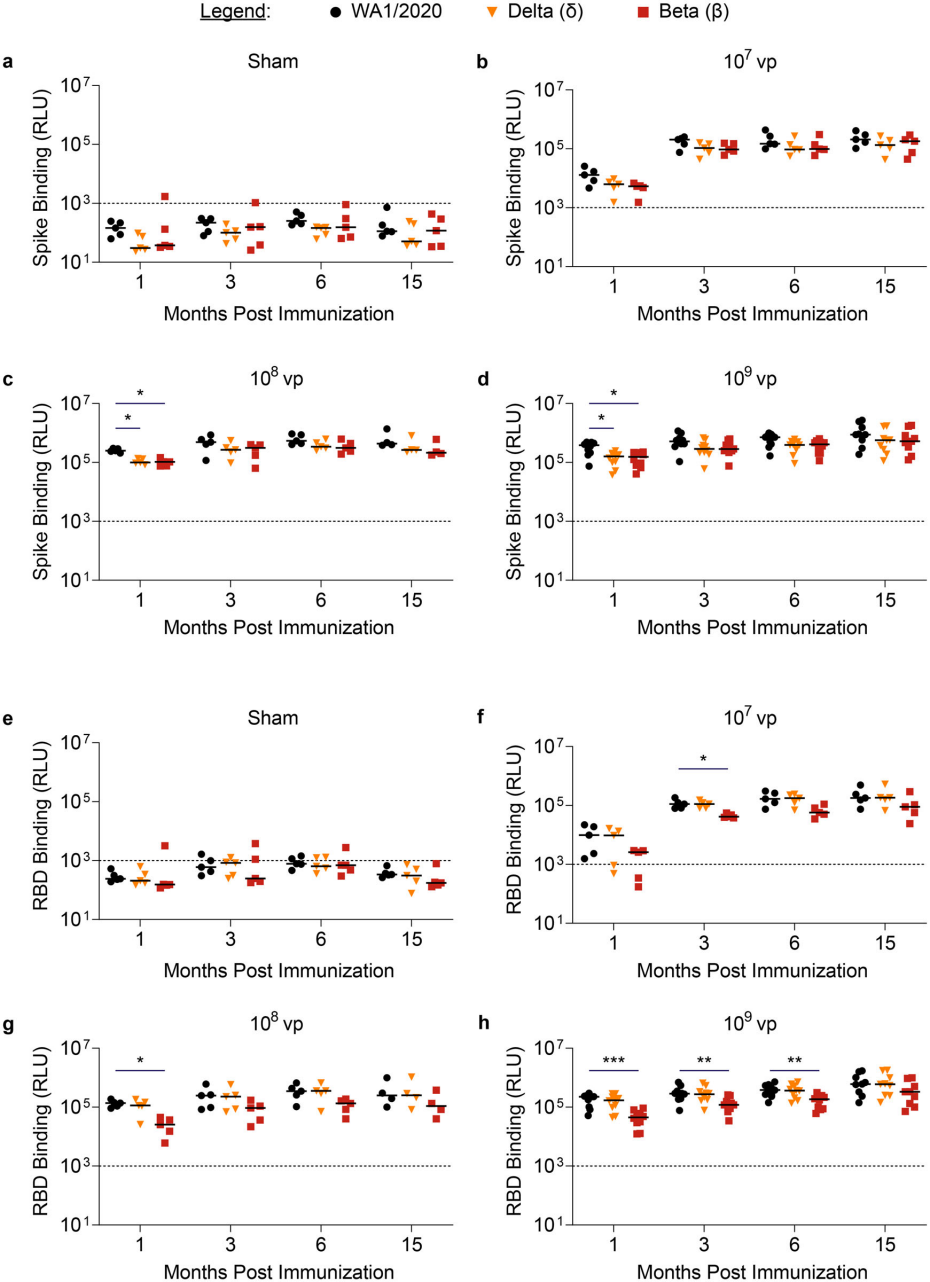
Binding antibody responses to variants expand in Ad26.COV2.S vaccinated mice. Spike-specific binding antibody responses to WA1/2020 (black circles), delta (yellow triangles), and beta variants (red squares) were assessed by electrochemiluminescence assays at months 1, 3, 6, and 15 postimmunization in (**a**) Sham-immunized (*N* = 5) or (**b**) 1 × 10^7^ vp (*N* = 5) (**c**) 1 × 10^8^ vp (*N* = 5) and (**d**) 1 × 10^9^ vp (*N* = 10) Ad26.COV2.S immunized mice. Similarly, receptor binding domain (RBD)-specific binding antibody responses to WA1/2020, delta, and beta variants were assessed by electrochemiluminescence assays at months 1, 3, 6, and 15 postimmunization in (**e**) Sham-immunized (*N* = 5) or (**f**) 1 × 10^7^ vp (*N* = 5) (**g**) 1 × 10^8^ vp (*N* = 5) and (**h**) 1 × 10^9^ vp (*N* = 10) Ad26.COV2.S immunized mice. Horizontal bars reflect median binding values. Dotted lines reflect assay limit of quantification. P values reflect Kruskal Wallis tests with Dunn’s multiple comparisons, which were conducted separately for each dose and timepoint (*P < 0.05; **P < 0.01; ***P < 0.001).

We next explored the kinetics of binding antibody responses to emerging SARS-CoV-2 variants of concern in the mice immunized with Ad26.COV2.S or sham. Peripheral blood was collected at the indicated timepoints and binding antibody responses were evaluated for WA1/2020, beta, and delta variant S protein and receptor binding domain (RBD) protein by an electrochemiluminescence assay (ECLA). As expected, sham-immunized mice exhibited median binding titers below the lower limit of quantification of the assay at all timepoints (Fig. 2a, e). Mice immunized with the low dose Ad26.COV2.S exhibited low but detectable binding titers to WA1/2020, delta, and beta variant S protein at 1 month postimmunization, which generally increased by month 3 and were sustained at month 15 (Fig. 2b). The middle and high doses elicited robust binding to WA1/2020 S by month 1, but significantly lower binding to delta variant S (middle dose *P* = 0.033; high dose *P* = 0.014) and beta variant S (middle dose *P* = 0.018; high dose *P* = 0.029). However, by month 3 and through month 15 postimmunization, S binding responses to all variants were high and statistically similar (Fig. 2c, d).

Regarding RBD binding, mice immunized with the low dose Ad26.COV2.S exhibited 1-month postimmunization responses near the lower limit of quantification of the assay (1000 RLU). Significantly lower binding to beta variant compared to WA1/2020 RBD was observed at 3 months postimmunization (*P* = 0.033), but not at 6 months and 15 months postimmunization (Fig. 2f). In mice immunized with the middle and high dose, 1 month postimmunization antibody binding to beta variant RBD was significantly lower than binding to WA1/2020 RBD (middle dose *P* = 0.033; high dose *P* = 0.0008). By month 15 postimmunization, RBD binding was statistically similar across all variants in both middle and high dose groups (Fig. 2g, h).

### Antibody neutralization of SARS-CoV-2 variants

**Fig. 3:**
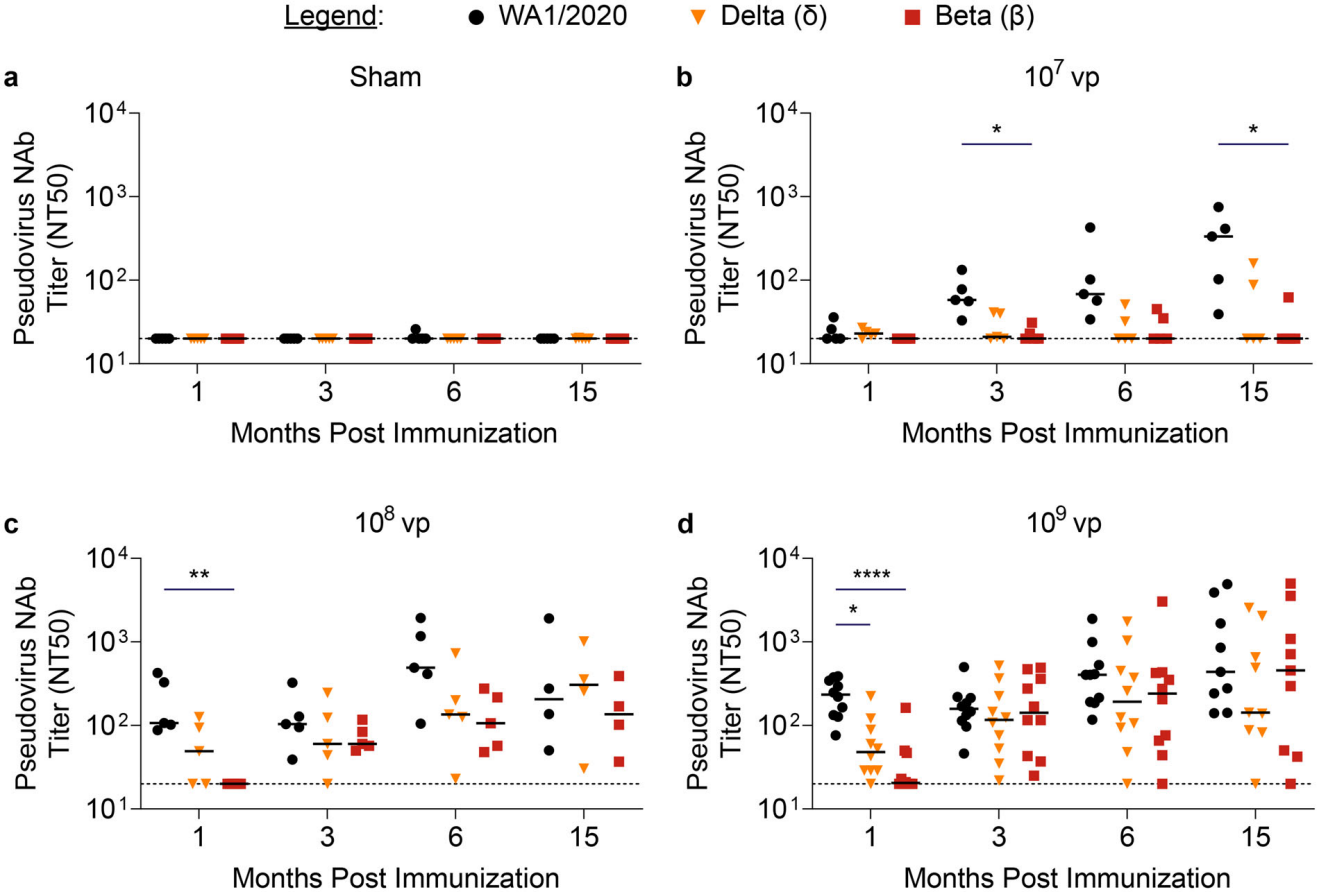
Neutralizing antibody responses to variants expand in Ad26.COV2.S vaccinated mice. Neutralizing antibody (NAb) responses to pseudoviruses expressing WA1/2020 (black circles), delta (yellow triangles), or beta variant (red squares) SARS-CoV-2 spike protein were evaluated at months 1, 3, 6, and 15 postimmunization in (**a**) Sham-immunized (*N* = 5) or (**b**) 1 × 10^7^ vp (*N* = 5) (**c**) 1 × 10^8^ vp (*N* = 5) and (**d**) 1 × 10^9^ vp (*N* = 10) Ad26.COV2.S immunized mice. Neutralizing responses are displayed as 50% neutralization titer (NT50). Horizontal bars reflect median NT50. Dotted lines reflect assay limit of quantification. *P* values reflect Kruskal Wallis tests with Dunn’s multiple comparisons, which were conducted separately for each dose and timepoint (*P < 0.05; **P < 0.01; ***P < 0.001; ****P < 0.0001).

To understand the NAb breadth induced by Ad26.COV2.S over time, an in-vitro pseudovirus neutralization assay was performed using pseudotyped viruses expressing the WA1/2020, delta, or beta variants of the SARS-CoV-2 spike protein. As expected, sham-immunized mice displayed undetectable virus neutralization at all timepoints (Fig. 3a). Mice that were immunized with low dose Ad26.COV2.S displayed NAb titers at or near the lower limit of detection across all variants at 1 month postimmunization, but detectable and increasing WA1/2020 pseudovirus NAbs in 5/5 mice by 3 months and through 15 months postimmunization. Conversely, the low dose variant pseudovirus NAbs were detectable in just 2/5 mice for delta and the same 2/5 mice for beta variants, with the latter significantly lower than WA1/2020 pseudovirus NAb titers at 3 months postimmunization (*P* = 0.015) and 15 months postimmunization (*P* = 0.022) (Fig. 3b).

The middle dose Ad26.COV2.S elicited robust neutralization of WA1/2020 pseudovirus, but diminished neutralization of delta variant pseudovirus with 3/5 mice displaying detectable NAb titers, and significantly reduced neutralization of beta variant pseudovirus (*P* = 0.007) with 0/5 mice displaying detectable NAb titers at 1 month postimmunization. Variant NAb titers were statistically similar by 3 months postimmunization, with all mice displaying detectable neutralization of delta and beta variant pseudoviruses through 15 months postimmunization (Fig. 3c). Similarly, the high dose Ad26.COV2.S induced robust neutralization of WA1/2020 pseudovirus at 1 month postimmunization, but significantly reduced neutralization of delta (*P* = 0.020), and beta (*P* < 0.0001) variant pseudoviruses was observed. By 3 months postimmunization, neutralization did not significantly differ across variants, and this trend persisted through 15 months postimmunization (Fig. 3d). Taken together, these data show that NAb responses to the SARS-CoV-2 beta and delta variant increased over time.

### Spike-specific antibody-secreting cells in spleen and bone marrow

**Fig. 4:**
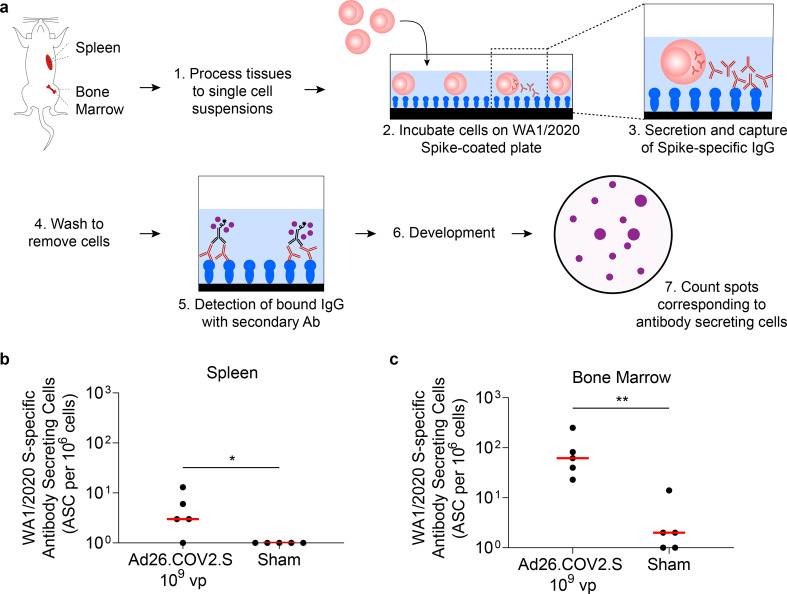
Antibody-secreting cells in Ad26.COV2.S vaccinated mice are present in spleen and bone marrow. The presence of antibody-secreting cells was assessed by ELISPOT at 15 months postimmunization in mice immunized with Ad26.COV2.S 1 × 10^9^ vp (*N* = 5) or Sham (*N* = 5). **a** Schematic illustrating the antibody-secreting cell ELISPOT experiment design in which cells were obtained from (**b**) spleen or (**c**) bone marrow and plated on wells coated with WA1/2020 full-length spike. Spot forming cells (SFCs) were normalized per 10^6^ cells plated. Horizontal bars reflect median SFC per 10^6^ cells. *P* values reflect two-sided Mann-Whitney tests (*P < 0.05; **P < 0.01).

Given the long-term durability of binding and neutralizing activity following a single immunization of Ad26.COV2.S, we sought to determine the source of circulating antibodies at 15 months postimmunization in mice that received the high vaccine dose compared to sham controls. An enzyme-linked immunospot (ELISPOT) assay was performed to detect and quantify WA1/2020 S-specific antibody-secreting cells collected from either the spleen or the bone marrow (Fig. 4a). At 15 months postimmunization, cells collected from the spleens of mice that were immunized with the high dose Ad26.COV2.S exhibited median WA1/2020 S-specific spot forming cells (SFC) of 3 per 10^6^ splenocytes. This result was higher than the negative control of splenocytes from sham-immunized mice (*P* = 0.048) (Fig. 4b). Bone marrow from high dose Ad26.COV2.S immunized mice exhibited WA1/2020 S-specific SFCs at a median frequency of 62 per 10^6^ cells. Similar to results in the spleen, this frequency was statistically significantly higher than the negative control of sham-immunized mice (*P* = 0.008) (Fig. 4c). In Ad26.COV2.S immunized mice, antibody-secreting cells that were bone marrow derived appeared greater in magnitude than those that were spleen derived.

## Discussion

In this study, we demonstrate superior immunogenicity of Ad26.COV2.S compared to Ad26.S.dTM.PP, a finding that extends previous data reported in mice^11^, hamsters^10^, and non-human primates^9,38^. The etiology of this observation may relate to differential processing and presentation of S.PP and S.dTM.PP following adenoviral cell entry^39^. Here, further preclinical evaluation of Ad26.COV2.S in mice showed that binding and neutralizing antibodies were durable for 15 months. Moreover, despite relatively small group sizes, a significant expansion in breadth of neutralizing antibody responses against SARS-CoV-2 variants over time was detected. The mechanistic basis for this expansion in neutralization breadth following vaccination requires further investigation, potentially with larger group sizes.

The recent emergence of several SARS-CoV-2 variants of concern poses the threat of viral escape from vaccine-induced polyclonal neutralizing antibodies or those raised by natural infection^16,17^. Despite the surge in cases and subsequent increase in hospitalizations following introduction of the delta variant to the United States, COVID-19 vaccines continue to display robust protection from severe disease^22^. Humoral responses have been implicated as an important correlate of protection^7–14^, although vaccine-elicited cellular immunity may also be mediating protection from severe disease^40–42^. Interestingly, it was recently shown that T-cell responses to variants are not impaired to the same degree as binding and NAb responses to variants in humans^31^. In convalescent COVID-19 patients, memory B-cell responses evolve up to 6–12 months following infection and produce antibodies with accumulating somatic mutations leading to expanded neutralization breadth and potency^43,44^. The expansion of neutralization breadth and increased magnitude of antibody titers over time observed in human patient samples^36^, as well as in this study in mice has largely not been reported with mRNA or other vaccine platforms. Whether these antibody dynamics are unique to Ad26.COV2.S or generalizable across other vaccine platforms remains an open question of active interest in both preclinical models and observational clinical studies^45^. Establishing a reliable animal model to further examine observations made in humans is therefore advantageous in building a nuanced understanding of immune responses following vaccination.

We have previously shown in humans that Ad26.COV2.S elicits durable cellular and humoral immune responses to emerging variants of concern 8 months after a single immunization^36^. The etiology of this observation is not well understood, although some studies have reported that early induction and persistence of germinal center B-cells^46^ and plasmablasts in the draining lymph nodes^47^, as well as the formation of long-lived plasma cells in the bone marrow^45,48^ and circulating memory B-cells in the peripheral blood^49^, may play a role. Indeed, the formation of vaccine-elicited memory B cells and long-lived plasma cells has classically been associated with durable, protective humoral immune responses in other disease models^50^. In line with these observations, our data point to the presence and persistence of WA1/2020 S-specific antibody-secreting cells in Ad26.COV2.S immunized mice at 15 months following immunization. In the bone marrow, WA1/2020 S-specific antibody-secreting cells appeared greater in magnitude than those found in the spleen, suggesting that formation of long-lived plasma cells, which are known to reside in the bone marrow^51,52^, is a likely source for circulating binding and neutralizing antibodies at the 15-month timepoint.

Pseudotyped viruses expressing the SARS-CoV-2 S protein were used to assess antibody neutralization, rather than live SARS-CoV-2 viruses. Prior work has established the pseudovirus platform and demonstrated strong positive correlations between pseudovirus and live virus NAb titers^7,9,11,53^. Future preclinical or clinical studies could further explore the observations made in the present study by probing the mechanisms underpinning the broad neutralization of variants. For example, experiments could assess the degree of somatic hypermutation occurring following vaccination or test whether broad neutralization is driven by few or many monoclonal antibodies with varying specificities. The data reported here serve as a foundation to further evaluate and probe the evolution of antibody responses that we have observed in humans in a high-throughput, tractable animal model. Moreover, a reliable mouse model could be harnessed to explore immunologic mechanisms through deeper analysis of several tissue compartments. Taken together, our data indicate that Ad26.COV2.S elicits durable expansion of neutralizing humoral responses to SARS-CoV-2 variants in mice and, more broadly, extend recently published data in humans, validating this animal model as a potential tool to further investigate immune responses following vaccination with Ad26.COV2.S.

## Materials and methods

### Animals and study design

Ad26 vectors expressing the S.PP or S.dTM.PP variant of the SARS-CoV-2 Spike protein sequence (Wuhan/WIV04/2019; GenBank MN996528.1) were constructed^9^. Briefly, replication-incompetent, E1/E3-deleted Ad26 vectors^33^ were generated in PER.C6.TetR cells using a plasmid containing the full Ad26 vector genome and a transgene expression cassette. Female BALB/c mice aged 6–8 weeks were randomly allocated to groups. Mice received either Ad26.COV2.S or Ad26.S.dTM.PP via the intramuscular (IM) route, titrated to doses of 1 × 10^7^ (*N* = 5), 1 × 10^8^ (*N* = 5), or 1 × 10^9^ (*N* = 10) viral particles (vp). Peripheral blood was drawn at the indicated timepoints. One mouse in the Ad26.S.dTM.PP low dose group died at 3 months postimmunization and was excluded from the analysis in Fig. 1. One mouse in the Ad26.COV2.S middle dose group died prior to the 15-month timepoint but was kept for the variant analysis at months 1, 3, and 6. At 15 months postimmunization, mice immunized with Ad26.COV2.S 1 × 10^9^ vp or sham-immunized mice were sacrificed and the spleen and bone marrow were collected for an antibody-secreting cell ELISPOT assay as described below. All animal studies were conducted in compliance with all relevant local, state and federal regulations and were approved by the BIDMC Institutional Animal Care and Use Committee.

### ELISA

WA1/2020 full-length spike (S) binding antibodies were assessed by ELISA. Briefly, plates were coated with 1 μg/ml of SARS-CoV-2 S protein (Sino Biological), diluted in 1 × PBS, and incubated at 4 °C overnight. After incubation, plates were washed once with a wash buffer (0.05% TWEEN-20 in 1 × PBS) and blocked with 350 μl of casein block per well. The block solution was discarded after 2–3 h of incubation at room temperature and plates were blotted dry. Three-fold serial dilutions of mouse serum in casein block were added to wells and plates were incubated for 1 h at room temperature. Plates were then washed three times and rabbit anti-mouse IgG HRP (Jackson ImmunoResearch Cat No: 315-035-045), diluted 1:1000 in casein block, was added to wells and incubated at room temperature in the dark. After 1 h, plates were washed three times, and 100 μl of SeraCare KPL TMB SureBlue Start solution was added to each well. Development was halted with the addition of 100 μl of SeraCare KPL TMB Stop solution per well. The absorbance at 450 nm was recorded using a VersaMax microplate reader. ELISA endpoint titers were defined as the highest reciprocal serum dilution that yielded an absorbance >0.2. The raw OD values were transferred into GraphPad Prism for analysis. A standard curve was interpolated using a sigmoidal four-parameter logistic (4PL) fit. To quantify the endpoint titer, the interpolation function was used to calculate the dilution at which the OD value would be equal to a value of 0.2.

### Pseudovirus neutralization assay

SARS-CoV-2 pseudoviruses were generated expressing a luciferase reporter gene^8,37^. Briefly, the packaging plasmid psPAX2 (AIDS Resource and Reagent Program), luciferase reporter plasmid pLenti-CMV Puro-Luc (Addgene), and spike protein-expressing pcDNA3.1-SARS CoV-2 SΔCT of variants were co-transfected into HEK293T cells by lipofectamine 2000 (ThermoFisher). Pseudoviruses of SARS-CoV-2 variants were generated for the WA1/2020 strain (Wuhan/WIV04/2019, GISAID accession ID: EPI_ISL_402124), B.1.617.2 (GISAID accession ID: EPI_ISL_2020950), and B.1.351 variant (GISAID accession ID: EPI_ISL_712096). The supernatants containing the pseudotype viruses were collected 48 h post-transfection, which were purified by centrifugation and filtration with 0.45 µm filter. To determine the neutralization activity of the plasma or serum samples from participants, HEK293T-hACE2 cells were seeded in 96-well tissue culture plates at a density of 1.75 × 104 cells/well overnight. Three-fold serial dilutions of heat-inactivated serum or plasma samples were prepared and mixed with 50 µL of pseudovirus. The mixture was incubated at 37 °C for 1 h before adding to HEK293T-hACE2 cells. 48 h after infection, cells were lysed in Steady-Glo Luciferase Assay (Promega) according to the manufacturer’s instructions. SARS-CoV-2 neutralization titers were defined as the sample dilution at which a 50% reduction in the relative light unit (RLU) was observed relative to the average of the virus control wells.

### Electrochemiluminescence assay (ECLA)

ECLA plates (MesoScale Discovery SARS-CoV-2 IgG Cat No: N05CA-1; Panels 11 and 13) were designed and produced with up to ten antigen spots in each well^38^. Briefly, the antigens included WA1/2020, delta, and beta variant spike and RBD proteins. The plates were blocked with 50 μL of Blocker A (1% bovine serum albumin (BSA) in distilled water) solution for at least 30 min at room temperature shaking at 700 rpm with a digital microplate shaker. During blocking, the serum was diluted 1:5000 in Diluent 100 (MesoScale Discovery). The plates were then washed three times with 150 μL of Wash Buffer (0.5% Tween in 1x PBS), blotted dry, and 50 μL of the diluted samples were added in duplicate to the plates and set to shake at 700 rpm at room temperature for at least 2 h. Secondary antibody was prepared using Jackson Immuno Rabbit Anti-Mouse IgG detection antibody (Cat No: 315-005-045) conjugated to the MSD GOLD SULFO-TAG by NHS-Ester chemistry per the manufacturer’s guidelines (Cat No: R91AO-1). The plates were again washed three times and 50 μL of tagged secondary antibody solution diluted 1:1000 in Diluent 100 was added to each well and incubated shaking at 700 rpm at room temperature for at least 1 h. Plates were then washed three times and 150 μL of MSD GOLD Read Buffer B was added to each well and the plates were read immediately after on a MESO QuickPlex SQ 120 machine. MSD titer for each sample was reported as Relative Light Units (RLU) which were calculated as average Sample RLU minus Blank RLU for each sample. The limit of detection was defined as 1000 RLU for each assay.

### Antibody-secreting cell ELISPOT

Detection of antigen-specific antibody-secreting cells was based on an assay previously reported by Shah et al.^54^. Briefly, Millipore Multiscreen HTS plates (Cat No: MSIPS4W10) were prewet with 15 µL of 35% ethanol per well for 1–2 min at room temperature. The plates were washed twice with 250 µL of PBS and then coated overnight at 4 °C with 100 µL per well of 5 µg/mL of WA1/2020 full-length spike (S) protein in PBS. The following day, the spleen and bone marrow of the mice were harvested and processed into a single cell suspension. Tissues were passed through a 70 µm filter into R10 media (RPMI + 10% FBS) to remove debris. Following centrifugation, the supernatant was discarded, and the pellet was resuspended in 2 mL ACK lysis buffer for 2 min to remove red blood cells. R10 media was added to stop the lysis reaction and bring the cell suspension to 20 mL. The suspension was centrifuged, the supernatant was discarded, and the pellet was resuspended in a final volume of 5 mL R10 media. The cells were counted, and the density was adjusted to 1 × 10^7^ cells per mL. The coated plates were washed twice with 250 µL of PBS and blocked at room temperature for 2 h with R10 media. Threefold serial dilutions of the cells were plated and incubated at 37 °C for 5 h. The plates were then shifted to 4 °C overnight. The following day the plates were washed 3 times with Wash Buffer (PBS + 0.05% Tween). 0.5 µg/mL of biotinylated Jackson Immuno Rabbit Anti-Mouse IgG detection antibody (Cat No: 315-065-045) in PBS + 5% FBS was added to the plates and incubated for 2 h at room temperature. WA1/2020 S-specific antibody-secreting cells were then detected using streptavidin-alkaline phosphatase conjugate and BCIP (5-bromo-4-chloro-3-indolylphosphate) substrate (Roche, Mannheim, Germany). Wells were imaged, and spot-forming cells (SFC) were counted by using a KS ELISPOT compact system (Carl Zeiss, Oberkochen, Germany). The number of SFCs per 10^6^ cells was calculated and reported.

### Statistical analyses

All statistical analyses were performed using GraphPad Prism version 9.1.2 (GraphPad Software). Comparison of two groups was performed using two-sided Mann-Whitney tests. Comparison of three groups or more was performed using Kruskal-Wallis tests with Dunn’s multiple comparisons. P values less than 0.05 were considered significant.

### Reporting summary

Further information on research design is available in the Nature Research Reporting Summary linked to this article.

## Supplementary information

REPORTING SUMMARY

## Data Availability

All data are available in the manuscript or from the corresponding author upon reasonable request.
